# Neuroimaging of Propofol Infusion Syndrome: A Case Report and Review of Literature

**DOI:** 10.7759/cureus.10583

**Published:** 2020-09-22

**Authors:** Elizabeth Pernicone, Pankaj Watal, Deeksha Dhar, Laura L Hayes, Tushar Chandra

**Affiliations:** 1 Medicine, University of Central Florida College of Medicine, Orlando, USA; 2 Pediatric Radiology, Nemours Children's Hospital, Orlando, USA; 3 Medicine, Government Medical College and Affiliated Hospitals, Jammu, IND; 4 Pediatric Radiology, Nemours Children's Clinic, Pensacola, USA

**Keywords:** neuroimaging, propofol infusion syndrome, anoxic injury, hypoxic injury, encephalopathy, pediatrics

## Abstract

A school-age boy with a complex medical history underwent a minor elective surgical procedure. Propofol was used for sedation during the procedure. The patient could not be awakened post-operatively. Laboratory findings demonstrated metabolic lactic acidosis, leukocytosis with bandemia, and transaminitis. Neuroimaging demonstrated findings that were consistent with hypoxic-ischemic or toxic-metabolic brain injury involving the bilateral basal ganglia, hippocampi, and cerebellum. The patient’s condition progressively worsened over the course of the following few weeks, and brain death was confirmed by scintigraphy seven weeks later. Prompt neuroimaging in unresponsive patients with suspected propofol infusion syndrome (PRIS) is of critical importance in detecting neurologic injuries, excluding alternative diagnoses, and determining prognostication.

## Introduction

Propofol infusion syndrome (PRIS) is a rare but serious complication of propofol infusion typically reported in seriously ill patients administered high doses of propofol for prolonged duration [1]. Classically, PRIS presents as acute refractory bradycardia, metabolic acidosis, and rhabdomyolysis, which may result in renal failure, cardiac failure, and death in the setting of propofol infusion [1]. Brain injuries or encephalopathy are uncommon manifestations of this syndrome but have been rarely reported [2,3]. Although the mechanism of PRIS is not yet well characterized, recent studies have demonstrated that propofol may inhibit key factors in the electron transport chain and in the transport of long-chain fatty acids, thus impairing beta oxidation of fatty acids and energy production [4-7]. In this case report, we detail neuroimaging findings in a pediatric patient who developed symptoms of PRIS.

## Case presentation

A school-age boy with history of prematurity, cerebral palsy, periventricular leukomalacia, and epilepsy underwent elective surgery for correction of strabismus. The patient's preexisting neurological conditions were well controlled at the time of surgery; he had normal developmental milestones and was fully alert and oriented with no neurological deficits before the procedure. He was premedicated with midazolam and anesthetized with propofol via continuous intravenous drip at a rate of 22 mcg/kg/min for 100 minutes. In addition to this continuous propofol drip, the patient received three bolus injections of propofol at a total dosage of 100 mg during the procedure. The surgical procedure was completed without any difficulty or excessive blood loss. The patient appeared to tolerate the surgery well with no significant abnormalities in vital signs noted during the procedure. He was briefly noted to be tachycardic with a heart rate of 148 beats per minute when first transported to the recovery area, but this finding resolved quickly and was unaccompanied by changes in other vital signs. No seizure activity was noted throughout this period. Unfortunately, failure of the patent to awaken was noted immediately afterwards, while still in the recovery room. Physical examination revealed extremity withdrawal to pain, but the patient was otherwise unresponsive. There were no vital sign abnormalities such as hypoxia, bradycardia, or hypotension during the patient’s surgery or during the post-operative period that were detected to explain the patient’s unresponsiveness. Likewise, the patient’s history did not reveal any exposure to known toxins. He did not have a history of mitochondrial disorder or other metabolic dysfunction. He was afebrile throughout the procedure and, other than an elevated white blood cell count noted immediately after the initial procedure, had no signs or symptoms of any sort of infection. Laboratory analyses following the procedure demonstrated metabolic acidosis, mildly elevated alanine aminotransferase (ALT), and an elevated white blood cell count with a left shift.

**Figure 1:**
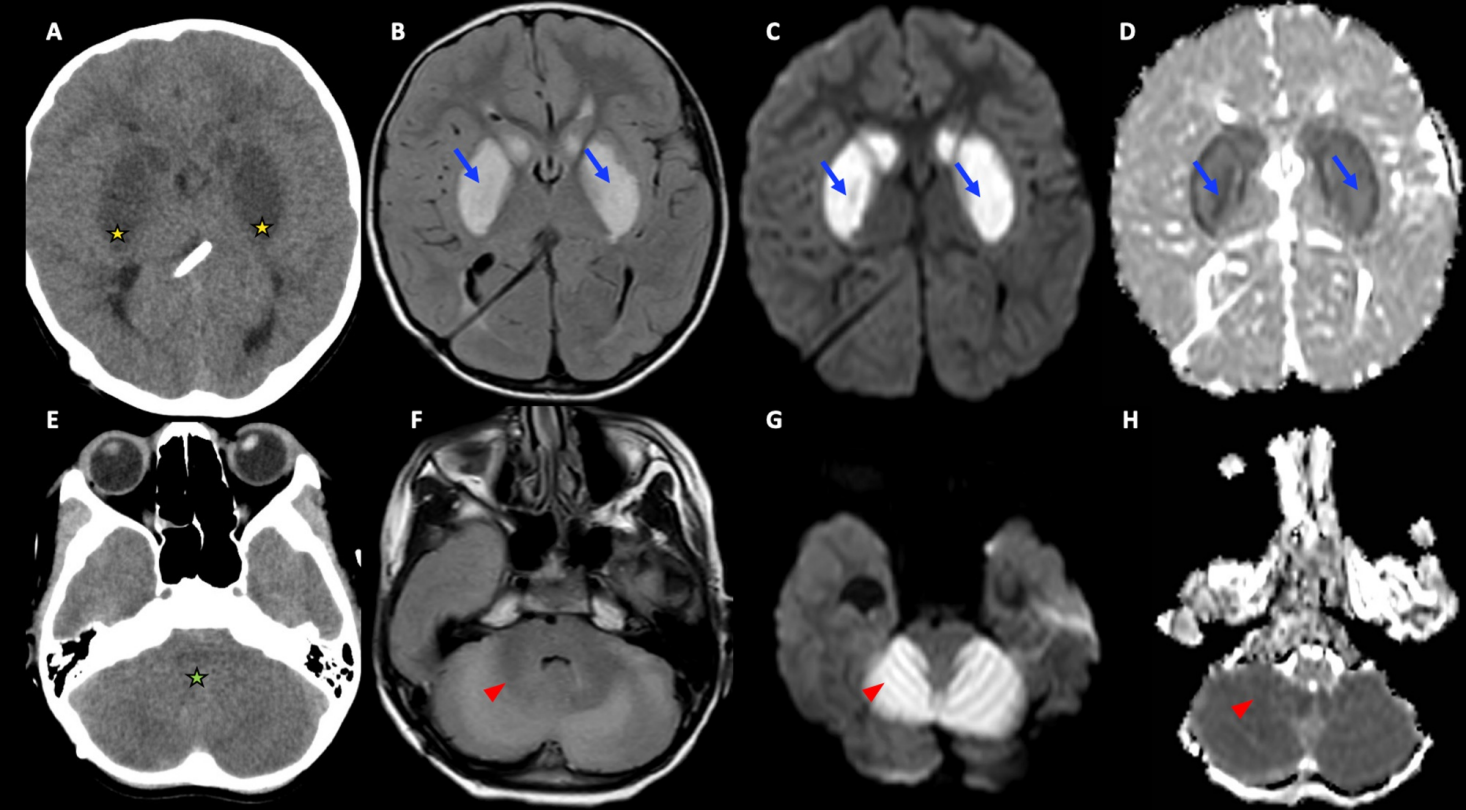
Initial CT and MRI Imaging Findings Images 1A and 1E: CT images demonstrated hypoattenuation in the basal ganglia (gold stars) and cerebellum (green star). Images 1B, 1C, 1D: T2 fluid-attenuated inversion recovery (FLAIR), isotropic diffusion-weighted, and apparent diffusion coefficient (ADC) map images demonstrated corresponding areas of diffusion restriction in the basal ganglia (blue arrows) suggestive of hypoxic-ischemic or toxic-metabolic injury. Images 1F, 1G, 1H: T2 FLAIR, isotropic diffusion-weighted, and ADC images demonstrated diffusion restriction in the cerebellum (red arrowheads) suggestive of hypoxic-ischemic or toxic-metabolic injury. These images were acquired immediately after the patient was noted to be unresponsive following his procedure.

An emergently acquired unenhanced CT scan of the patient’s brain demonstrated bilaterally symmetrical areas of hypoattenuation in the basal ganglia, mesial temporal lobes, midbrain, and cerebellum (Figure 1). No definite area of acute hemorrhage was identified. There was no midline shift. MR imaging of the brain without contrast was subsequently obtained and demonstrated extensive restricted diffusion with corresponding T2 and fluid-attenuated inversion recovery (FLAIR) hyperintense signal changes in bilateral basal ganglia, hippocampi, cerebral peduncles, and most of the cerebellum (Figure 1). There was significant mass effect on the fourth ventricle without any significant upstream dilatation of the ventricular system. No evidence of periventricular cerebrospinal fluid (CSF) seepage was noted. Following these findings, the patient was admitted to the pediatric intensive care unit (PICU) for further treatment and care.

**Figure 2:**
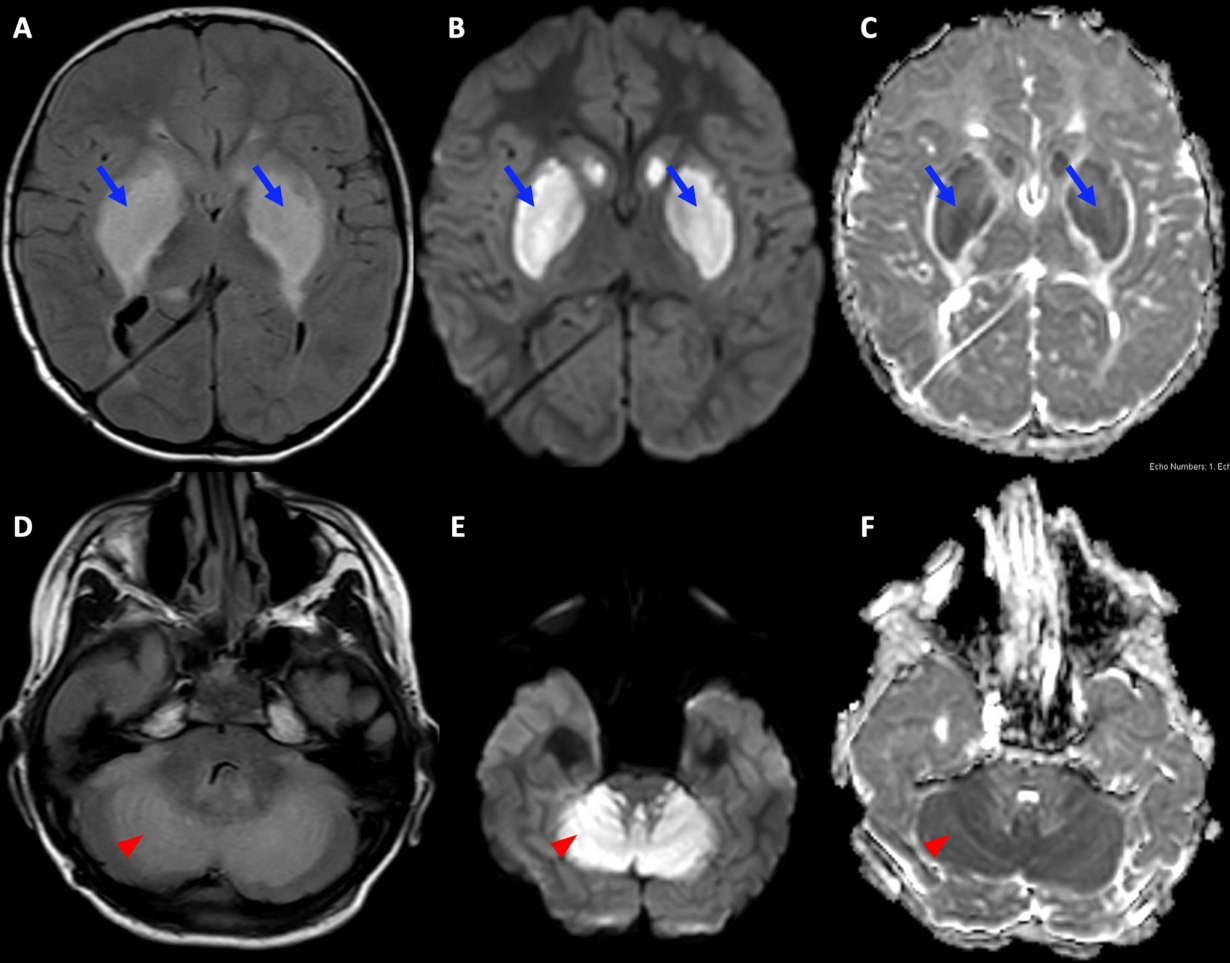
Follow-Up MRI Findings Obtained Three Days After Initial Imaging Images 2A, 2B, 2C: T2 fluid-attenuated inversion recovery (FLAIR), isotropic diffusion-weighted, and apparent diffusion coefficient (ADC) map images demonstrated increased areas of diffusion restriction in the basal ganglia (blue arrows). Images 2D, 2E, 2F: T2 FLAIR, isotropic diffusion-weighted, and ADC map images demonstrated increased areas of diffusion restriction in the cerebellum (red arrowheads).

Unfortunately, the patient’s condition continued to deteriorate with complete loss of response to all stimuli, and he required ventilatory support as he stopped making spontaneous respiratory efforts. A follow up MRI obtained three days after the initial surgical procedure demonstrated interval worsening of the cytotoxic edema involving bilateral basal ganglia, hippocampi, cerebral peduncles, and the cerebellum (Figure 2). In addition, interval development of cerebellar tonsillar herniation and ischemic changes in the upper cervical spinal cord were revealed. Time-of-flight MR angiogram (MRA) and MR venogram (MRV) of the brain were normal.

**Figure 3:**
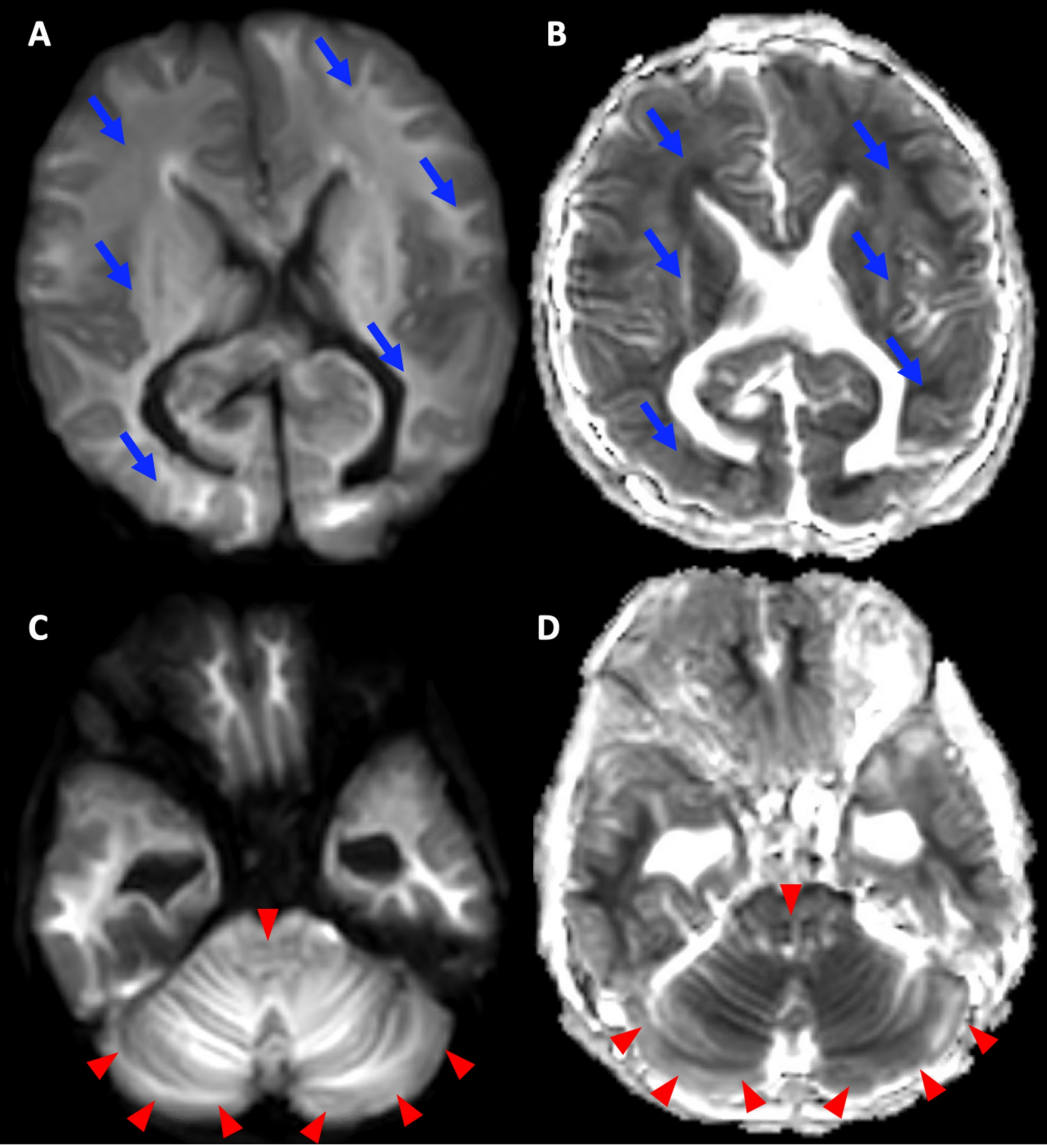
Follow-Up MRI Obtained One Month After Initial Imaging Image 3A, 3B: Isotropic diffusion-weighted image and apparent diffusion coefficient (ADC) maps demonstrated new, widespread diffusion restriction throughout the cerebral white matter (blue arrows). Images 3C, 3D: Isotropic diffusion-weighted image and ADC map demonstrated demonstrate new, widespread diffusion restriction in the cerebellum (red arrowheads).

**Figure 4:**
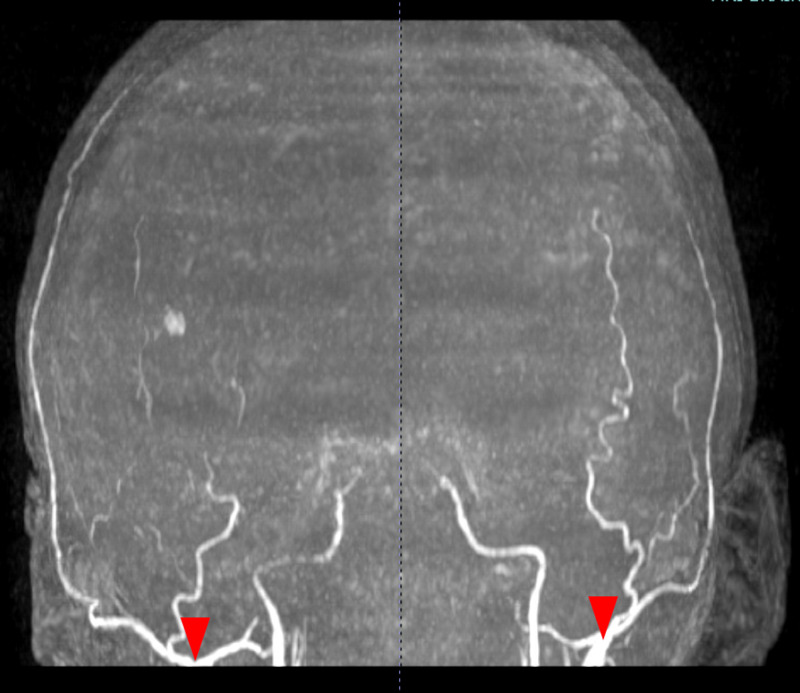
Time-of-flight MRA Obtained One Month After Initial Imaging Time-of-flight MR angiogram (MRA) one month after the patient was initially noted to be unresponsive showed absence of flow-related signal in the circle of Willis and branch arteries bilaterally. Red arrowheads indicate the distal internal carotid arteries with a lack of intracranial arterial flow.

Although at this point the patient’s clinical presentation indicated possible brain death, a reliable clinical brain death exam was not feasible on account of cervical cord ischemia. Supportive care of the patient was continued in the PICU. Subsequent MR imaging obtained four weeks later demonstrated persistent diffuse cytotoxic edema involving both cerebral hemispheres and the entire cerebellum. Further worsening of cerebellar herniation and persistent cervical cord ischemia were also evident. Time-of-flight MRA revealed absence of flow-related signal in the circle of Willis or any of its branches, with blooming identified in proximal bilateral middle cerebral arteries, proximal bilateral posterior cerebral arteries, and the basilar artery concerning for widespread arterial thrombosis (Figure 3). MRV showed absence of flow-related enhancement in the superior sagittal sinus, the straight sinus, bilateral transverse sinuses, and bilateral sigmoid sinuses, with corresponding hyperintense signal on T2 weighted imaging, concerning for widespread venous thrombosis (Figure 4).

**Figure 5:**
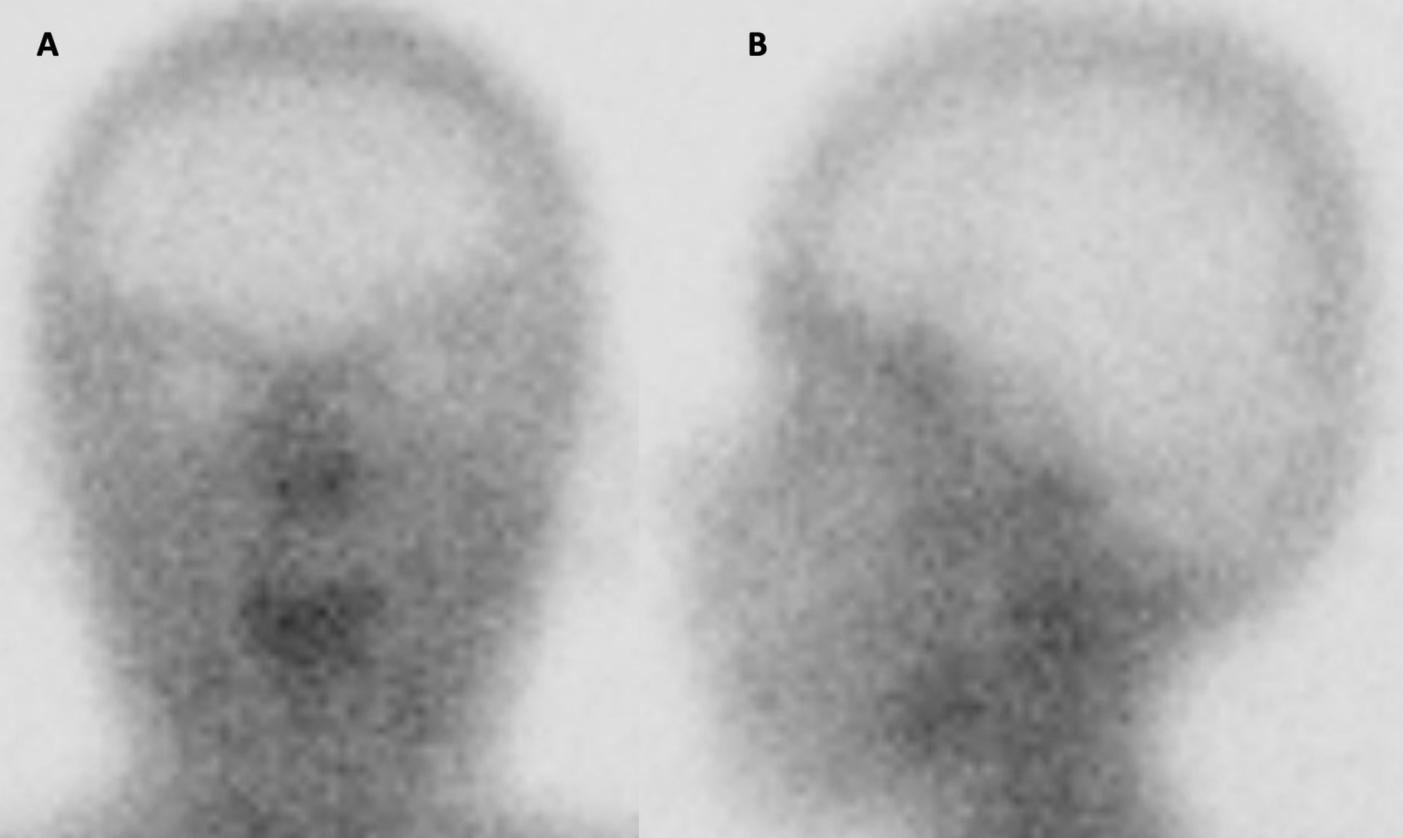
Nuclear Flow Study Obtained at Seven Weeks After Initial Procedure Anterior (A) and left lateral (B) static images acquired 15 minutes after intravenous injection of 99mTc hexamethyl propylene amine oxime (99mTc-HMPAO) demonstrate absence of brain perfusion (empty light bulb or hot nose sign). This finding is consistent with brain death in a relevant clinical setting, as observed in this patient.

A nuclear brain perfusion study performed one week later demonstrated diffuse absence of parenchymal radiotracer uptake, consistent with absence of brain perfusion (Figure 5). Given his failure to awaken after the procedure, the history of recent propofol infusion, immediate post-operative lab findings showing metabolic lactic acidosis, mild elevation of ALT, and the absence of other signs or symptoms pointing to another cause of these ailments; the patient was diagnosed with PRIS.

## Discussion

Propofol is a small lipophilic molecule with the ability to easily diffuse across mitochondrial membranes [7]. PRIS is a rare but serious complication of high dose and/or prolonged propofol administration reported in both adults and children. The exact incidence of PRIS is not known, partially due to the fact that its presentation is variable and it is, in most cases, a diagnosis of exclusion. At least 44 pediatric cases have been reported in the literature based on a recently published review [1]. At least one of the reported cases was observed after a single dose of propofol in a patient with underlying mitochondrial disease [1,8]. The updated definition of PRIS suggested by Hemphill et al. described PRIS as a syndrome characterized by unexplained metabolic acidosis, rhabdomyolysis, or electrocardiogram changes, with or without acute kidney injury, hyperkalemia, hyperlipidemia, cardiac failure, fever, elevated liver enzymes, or elevated lactate [1]. The average mortality rate in children is estimated at 52%, with fever and hepatomegaly suggested as indicators of mortality in the pediatric population [9].

Brain injuries or encephalopathy are rarely observed in this syndrome but have been reported [2,3]. Risk factors for PRIS include young age, severe concurrent illness, high fat or low carbohydrate intake, hypoglycemia, coadministration of catecholamine or steroid use, fatty acid oxidation disorders, or high doses of propofol (>4-5 mg/kg/hr) for a prolonged period (>48 hr) [1,5,9,10]. It must be noted that our patient was not genetically tested for inborn errors of metabolism that may have predisposed him to developing PRIS. While there are no specific protocols for management of PRIS, treatment typically includes immediate discontinuation of propofol, correction of pH and electrolyte imbalances, supportive care including extracorporeal membrane oxygenation when necessary, and hemodialysis in select cases where electrolyte or pH imbalances cannot be corrected via other means [11,12]. At this time, given the risks associated with prolonged propofol use in young patients, propofol is not currently recommended for long term sedation in pediatric patients [1].

The mechanism of PRIS is proposed to be propofol induced disruption of intracellular adenosine triphosphate (ATP) production, by interference in the mitochondrial electron transport chain or by interference with oxidative metabolism of free fatty acids within mitochondria. Most reported pediatric cases have been observed after continuous infusion of the drug [5,8]. In a small percentage of pediatric patients, severe PRIS-like syndrome after low dose propofol is suspected to be due to enhanced sensitivity from underlying mitochondrial defects. The clinical syndrome in these patients may resemble an acute presentation of patients with genetic mitochondrial diseases. Some authors suggest that close attention to maintaining a normoglycemic status in these patients may help to prevent development of PRIS [8].

Propofol is hypothesized to block the transport proteins in the mitochondrial membrane responsible for influx of long-chain fatty acids, thereby impairing beta oxidation of fatty acids [1,3,4]. Histopathological examination of skeletal and cardiac muscle in PRIS has demonstrated extensive myocyte necrosis and fat accumulation in some cases [5]. Previous studies have also proposed that propofol may block the electron transport chain through inhibition of Complex 1 or Coenzyme Q, thereby inhibiting electron transfer on Cytochrome C [1,5,6]. This pathogenic mechanism helps to explain why, in our patient and in another patient with documented brain injury due to PRIS, regions of the brain with relatively high metabolic activity-the basal ganglia, hippocampi, and cerebellum-demonstrated injury first.

Overall, similar acute findings have been reported on brain imaging after hypoxic brain injury in the setting of carbon monoxide toxicity, including spatial distribution [13-15]. This is not unexpected since both carbon monoxide and propofol action are centered on the mitochondrial electron transport chain, with carbon monoxide targeting complex IV (unlike complex I inhibition by propofol) [16]. Prompt neuroimaging in suspected PRIS patients may allow faster detection and prompt initiation of neuroprotective measures, and it may also predict prognosis. In our patient and in another patient whose basal ganglia showed restricted diffusion on MRI, the prognosis was very poor [2]. In the case reported by Li et al., imaging demonstrated extensive diffusion restriction in the basal ganglia and cerebellum similar to our patient, except that Li et al. also described hemorrhage of the left caudate nucleus that was observed on follow-up imaging. In contrast to our patient and the case described by Li et al., one other pediatric patient with reported brain involvement that spared the basal ganglia reportedly made an excellent recovery and regained previous function [3]. The pertinent details of these cases are compared in Table 1.

**Table 1:** Review of PRIS Literature Where Relevant Neuroimaging Was Available, Compared to Current Report PRIS: propofol infusion syndrome; DNA: deoxyribonucleic acid; FLAIR: fluid-attenuated inversion recovery; DWI: diffusion‐weighted imaging; ALT: alanine aminotransferase.

	Poretti et al. [2]	Li et al. [3]	Current case report
Age of Patient	3 year-old female	30 year-old female	6 year-old male
Risk factors	Young age	None	Young age
Genetic mitochondrial condition	Not mentioned	No abnormalities in mitochondrial DNA	Not tested
Indication for propofol use	Sclerotherapy of large venous malformation	Laparoscopic surgery for colon polyp/ cancer	Surgical repair of bilateral optic esotropia
Propofol rate and dose	Not available	Mean rate of 3.3 mg/kg/hr totaling 120 mg in 6 hours	Mean infusion rate of 1.32 mg/kg/hr for 100 min, plus 3 bolus doses totaling 100 mg in same time period
Imaging Findings (CT and MRI)	T2 prolongation and diffusion restriction was noted in supra- and infratentorial white matter; these findings were completely resolved on follow up.	Low attenuation was shown in bilateral basal ganglia, on CT. Follow up CT demonstrated hemorrhage in caudate nucleus. Diffusion signal abnormality of the basal ganglia, temporal lobe, and cerebellum bilaterally and a corresponding T1 hypointensity was also noted.	Low attenuation was evident in bilateral basal ganglia and cerebellum on CT. Diffusion restriction of bilateral basal ganglia, cerebellum, and bilateral hippocampi with hyperintensities was noted on T2, FLAIR, and DW MRI sequences. Four weeks later, diffuse cytotoxic edema on DWI was noted, with widespread hyperintensity on T2 and FLAIR sequence, involving the entire cerebral hemispheres and the cerebellum.
Laboratory Changes	Increased levels of acetyl and hydroxy-butyryl ketones with generalized elevation of fatty acylcarnitine intermediates.	N/A	Lactic acidosis; also, elevated leukocyte count with a left shift; mildly elevated ALT
Clinical outcome	Complete recovery	Death	Death

Although MR imaging was performed in cases reported by Savard et al. and Mtaweh et al., this was done for other indications well before the development of PRIS [6,8]. Similar to our case, Mtaweh et al. reported PRIS-like condition in a child caused by a single dose of propofol (1.25 mg/kg). In contrast, the case described by Savard et al. involved the development of PRIS in an adult at much higher doses administered for a longer period of time. Both case reports were associated with mitochondrial disorders or gene defects. Our patient had not previously experienced symptoms of any sort of mitochondrial disorder and was therefore never genetically tested for it.

## Conclusions

PRIS is a rare but potentially fatal complication of propofol infusion. Very little is known about possible neurologic sequelae resulting from PRIS. Knowledge of the imaging findings of PRIS is helpful for establishing more standardized diagnostic and management guidelines in patients with suspected PRIS so that more common injury patterns associated with this syndrome may be identified. Furthermore, awareness about this lethal complication is important to enable urgent neuroimaging for obtunded or unresponsive patients after administration of propofol.
